# Structure of the SOCS4-ElonginB/C Complex Reveals a Distinct SOCS Box Interface and the Molecular Basis for SOCS-Dependent EGFR Degradation

**DOI:** 10.1016/j.str.2007.09.016

**Published:** 2007-11-13

**Authors:** Alex N. Bullock, Maria C. Rodriguez, Judit É. Debreczeni, Zhou Songyang, Stefan Knapp

**Affiliations:** 1 University of Oxford, Structural Genomics Consortium, Botnar Research Centre, Oxford OX3 7LD, United Kingdom; 2 Verna and Marrs McLean Department of Biochemistry and Molecular Biology, Baylor College of Medicine, One Baylor Plaza, Houston, TX 77030, USA

**Keywords:** PROTEINS, SIGNALING

## Abstract

Tyrosine kinase signaling is tightly controlled by negative feedback inhibitors including suppressors of cytokine signaling (SOCS). SOCS assemble as SH2 domain substrate recognition modules in ElonginB/C-cullin ubiquitin ligases. In accordance, SOCS4 reduces STAT3 signaling from EGFR through increased receptor degradation. Variable C-termini in SOCS4–SOCS7 exclude these family members from a SOCS2-type domain arrangement in which a strictly conserved C terminus determines domain packing. The structure of the SOCS4-ElonginC-ElonginB complex reveals a distinct SOCS structural class. The N-terminal ESS helix functionally replaces the CIS/SOCS1–SOCS3 family C terminus in a distinct SH2-SOCS box interface that facilitates further interdomain packing between the extended N- and C-terminal regions characteristic for this subfamily. Using peptide arrays and calorimetry the STAT3 site in EGFR (pY^1092^) was identified as a high affinity SOCS4 substrate (K_D_ = 0.5 μM) revealing a mechanism for EGFR degradation. SOCS4 also bound JAK2 and KIT with low micromolar affinity, whereas SOCS2 was specific for GH-receptor.

## Introduction

Secreted cytokines and growth factors modulate the survival, proliferation, and differentiation of most cell types through type-specific receptor binding and activation of intracellular signaling cascades. Ligand-induced receptor dimerization activates intrinsic receptor tyrosine kinase (RTK) activity and for most cytokine receptors recruitment of Janus kinase/signal transducer and activator of transcription (JAK/STAT) (Ihle, 1995). A characteristic feature of receptor activation is the creation of phosphotyrosine docking sites for Src-homology 2 (SH2) domain proteins that modulate the intracellular response (Moran et al., 1990). The suppressor of cytokine signaling (SOCS) family was first recognized as a group of cytokine-inducible SH2 (CIS) domain proteins comprising eight family members in human (CIS and SOCS1–SOCS7) (Hilton et al., 1998; Starr et al., 1997). The four prototypical members, CIS and SOCS1–SOCS3, have been studied extensively and in a classic negative feedback response compete for binding at phosphotyrosine sites in JAK kinase and receptor pathways to displace effector proteins and target bound receptors for proteasomal degradation (Kile et al., 2002). Loss of SOCS activity results in excessive cytokine signaling associated with a variety of hematopoietic, autoimmune, and inflammatory diseases and certain cancers (O'Sullivan et al., 2007). Surprisingly, the remaining SOCS family members SOCS4–SOCS7, which represent the direct orthologs of ancestral SOCS family genes show limited cytokine induction, and it is of current interest to address their role in cytokine suppression and other signaling events.

The absence of a JAK kinase in *C. elegans* has suggested that its ancestral SOCS gene might fulfill a wider role regulating receptor tyrosine kinases (Kile et al., 2002). Several studies have demonstrated SOCS regulation of the epidermal growth factor receptor (EGFR or ErbB) family (Goldshmit et al., 2004; Kario et al., 2005; Nicholson et al., 2005; Rawlings et al., 2004; Xia et al., 2002). EGF signaling is a major determinant of epithelial cell proliferation, and due to its high oncogenic potential and incidence in cancer, the EGFR is one of the best characterized substrates for SH2 interactions (Citri and Yarden, 2006; Jones et al., 2006; Schulze et al., 2005). EGFR signaling is mediated either by direct STAT SH2 binding and transactivation or by the SH2 adaptor proteins Grb2 and Shc, which couple to the Ras-MAPK and Ras-PI3K-AKT/PKB pathways. Additional SH2 domain proteins confer downregulation, including the SHP1 phosphatase and the Cbl ubiquitin ligase, which directs EGFR degradation. A number of combinatorial control systems have evolved that lead to EGFR degradation in response to different stimuli (Citri and Yarden, 2006).

A role for SOCS in EGFR signaling has been suggested from studies in *Drosophila*, which presents a simplified model system with only one JAK, STAT, and EGFR family member (Arbouzova and Zeidler, 2006). Of the three *Drosophila* SOCS genes, only SOCS36E, a close ortholog of human SOCS4 and SOCS5, has shown prototypical SOCS negative feedback activity. Transgenic flies overexpressing SOCS36E display wing defects that phenocopy *Drosophila* mutants of JAK, STAT, and EGFR and are exacerbated in flies heterogeneous for these genes. Conversely, the defects are partially rescued by inactivating one copy of the d-*cbl* gene (Callus and Mathey-Prevot, 2002; Rawlings et al., 2004). Human SOCS4/SOCS5 share 90% sequence identity within the SH2 domain and 72% with the SOCS36E SH2 domain and conserved function in humans has been suggested by two recent studies showing SOCS4/SOCS5 regulation of EGFR signaling (Kario et al., 2005; Nicholson et al., 2005). In accordance with the classical SOCS model, EGF-induced expression of SOCS4 and SOCS5 reduced STAT3 signaling as a result of increased EGFR degradation. Expression of other SOCS family members did not produce this effect.

However, a genomic screen of recombinant SH2 domains failed to identify a significant SOCS-EGFR interaction (Jones et al., 2006) and further characterization of this interaction in vitro is currently lacking. Notably, 13 of the 14 SH2 domains tested from the STAT and SOCS families were expressed in inclusion bodies and refolding had limited success (Jones et al., 2006). We recently presented a general strategy to overcome this problem by coexpressing a multidomain SOCS construct with its constitutive binding partners ElonginB and ElonginC (Bullock et al., 2006). Using this approach, we were able to determine the domain organization of a SOCS family member with the crystal structure of the SOCS2-ElonginC-ElonginB complex (Bullock et al., 2006). The structure defines a prototypical SOCS box ubiquitin ligase. First, the SOCS box is conserved with the BC box of VHL, which also binds ElonginB/ElonginC and targets hypoxia-inducible factor (HIF-1α) for proteasomal degradation (Stebbins et al., 1999). Second, the positions of the substrate binding sites in SOCS2 (SH2) and VHL (β-domain), which are functionally unrelated, are superimposable within the ternary complexes, suggesting a common spatial requirement for ubiquitination.

An essential requirement of this model is a stable interface between the substrate binding domain and SOCS box. In SOCS2, the three helices of the SOCS box make no contact with the SH2 domain, and instead the C terminus occupies the interdomain interface, allowing the carboxy group to participate in a critical hydrogen-bonding network. This packing precludes C-terminal extensions and explains the strictly conserved length of the C terminus in CIS and SOCS1–SOCS3. However, this solution raised the question how members of the extended SOCS family would function and interact with elongins. The ancestral SOCS proteins and their human orthologs cannot adopt the same stable C-terminal packing since SOCS4, SOCS5, and SOCS7 show variable C-terminal extensions, while SOCS6 has a two residue truncation. To determine the alternative domain organization of this second SOCS subfamily and to understand the structural basis for EGFR degradation, we determined the structure of the SOCS4-ElonginB/C complex. A novel SOCS box interface is revealed that frees the extended C terminus to form a new interface stabilizing the N-terminal domain. To address the limited knowledge of SOCS substrate specificity we also characterized the binding of SOCS2 and SOCS4 to a degenerate peptide library as well as to known SOCS target sites. We observed strong submicromolar binding of SOCS4 to phosphotyrosine sites with +1 isoleucine including EGFR pY^1092^ providing a molecular mechanism for SOCS4 inhibition of STAT3 signaling as well as EGFR degradation.

## Results

**Figure 1:**
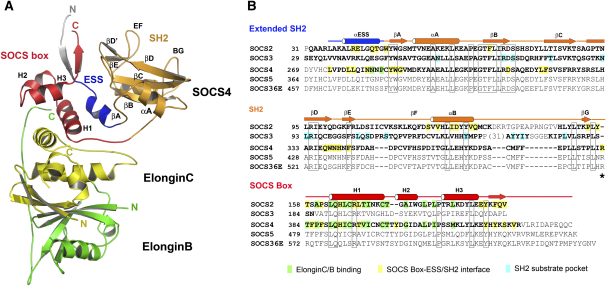
Structure of the SOCS4-ElonginC-ElonginB Ternary Complex and Structure-Based Sequence Alignment (A) The different SOCS4 domains are highlighted by color with the N-terminal ESS shown blue, the SH2 shown orange, and the SOCS box shown red. A structured region from the N-terminal hexahistidine tag that was protected from proteolytic cleavage in solution is colored gray. The other complex components ElonginC and ElonginB are colored yellow and green, respectively. (B) Secondary structure elements in SOCS4 are shown above the sequence alignment. Structure-determined residues from SOCS2–SOCS4 are shown in bold; unstructured insertions and homologous SOCS sequences are gray, while conserved residues are boxed. An asterisk marks the insertion of R383 in the SOCS4 hinge, which stabilizes the SOCS4 domain organization. This and other interface residues are highlighted by different colors in the alignment.

Human SOCS4 was coexpressed with its binding partners ElonginB and ElonginC in *E. coli*. The crystal structure of the corresponding SOCS4-ElonginC-ElonginB ternary complex was solved by molecular replacement and was refined to 2.5 Å resolution (see Table 1 for data collection and refinement statistics). Comparison of the SOCS2 (Bullock et al., 2006) and SOCS4 ternary complexes revealed a common tripartite domain structure with an N-terminal extended SH2 subdomain (ESS helix) that stabilizes the central SH2 domain and a C-terminal SOCS box that mediates a conserved four helix bundle interaction with ElonginC (Figure 1). SOCS4 has a two-residue insertion in this motif that extends the H2 3^10^-helix found in SOCS2. The C terminus of ElonginB packs beneath this helix and completes its hydrophobic packing.

### The Extended SH2 Domain Is a Conserved Structural Element

**Figure 2:**
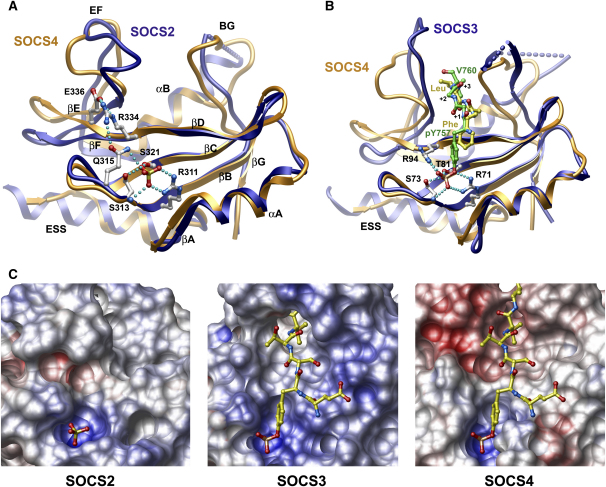
Structural Comparison of the SH2 Substrate Pockets in SOCS2, SOCS3, and SOCS4 (A) Overlay of the crystal structures of the SOCS4 SH2 (orange) and the SOCS2 SH2 (blue, PDB code: 2C9W). The binding site of the phosphotyrosine moiety in SOCS2 is indicated by the presence of a bound sulfate ion. SOCS4 residues at this site are shown in ball-and-stick representation with their potential hydrogen bonding. (B) Overlay of the crystal structures of the SOCS4 SH2 (orange) and the murine SOCS3 gp130 complex (blue, PDB code: 2HMH). The phosphotyrosine and three C-terminal residues from the murine gp130-derived peptide are colored green. N-terminal tag residues from a crystallographic SOCS4 neighbor occupy the same SH2 pocket in the SOCS4 structure forming substrate mimetic interactions and are colored yellow. (C) Surface representation of the SH2 substrate pocket in SOCS2, SOCS3, and SOCS4 colored by electrostatic potential. The bound sulfate ion identifies the phosphotyrosine binding site in SOCS2 (left). The gp130 peptide is shown in complex with SOCS3 (center) and docked onto the SOCS4 surface (right) by the overlay of the two structures (for SOCS4 only the gp130 residues corresponding to the pY−1 to pY+3 positions are shown).

SOCS proteins possess a highly variable N-terminal domain with no homology to known structural domains. Mutagenesis studies identified an ESS helix in CIS and SOCS1–SOCS3 that was critical for high affinity SH2 substrate interactions (Yasukawa et al., 1999) and an additional N-terminal kinase inhibitory region (KIR domain) resembling a JAK pseudosubstrate in SOCS1 and SOCS3 (Yasukawa et al., 1999; Nicholson et al., 1999). In the structure of SOCS4, the ESS forms a single helix N-terminal to the SH2 domain that packs behind the BC and DE β-hairpins. This structure has not been described previously for SOCS4–SOCS7 but appears to be a general feature of the SOCS SH2 domain. Notably, the ESS and SH2 domains are superimposable as a single structural motif in SOCS2 (Bullock et al., 2006), SOCS3 (Babon et al., 2006; Bergamin et al., 2006), and SOCS4 (Figure 2). These extended SH2 domains are stabilized by a conserved hydrophobic surface on the ESS that buries residues from βA, βB, βC, βE, and αA. SOCS4 is distinguished by a proline insertion that restricts the length of the ESS to half that of the SOCS2 and SOCS3 helices and corresponds to the loss of structure in the equivalent KIR domain region. Interestingly, this shorter ESS is highly similar to the N-terminal helices associated with the Cbl (αN) (Meng et al., 1999) and STAT family SH2 domains (e.g., STAT1 α11) (Chen et al., 1998) which fulfill a similar packing role.

The SOCS4 SH2 domain adopts a canonical SH2 fold with the substrate pocket positioned on the opposite face to the ESS. SH2 ligands bind across the central βD strand which separates the phosphotyrosine (pY) pocket from the hydrophobic +3 site where ligand specificity is typically determined. The overall structure of the phosphotyrosine pocket in SOCS4 is similar to SOCS2 and SOCS3, but the pocket side chains adopt different roles binding the phosphotyrosine ligand (Figure 2A). CIS and SOCS1–SOCS3 are characterized by the absence of the common SH2 αA2 arginine, and bind phosphotyrosine instead with two conserved arginine residues at the βB5 and βD6 positions (Figure 2B) (SOCS3 R71 and R94, respectively). The SOCS4 SH2 domain harbours a lysine residue at the αA2 position, which also ligates the phosphotyrosine in STAT1 (Mao et al., 2005). The SOCS4 βD6 arginine (R334) is also distinguished by forming a hydrogen bond network with Q315 and E336 that removes the arginine side chain from the phosphotyrosine binding site. However, the βD6 interaction is likely to be substituted by Q315 Nɛ, which occupies the same position as R344 Nη in the SOCS2/SOCS3 structures (Figure 2A). Interestingly, the SOCS4 substrate binding pocket is occupied by residues from the N-terminal tag sequence of a crystallographic neighbor (Figure 2B). The main chain follows closely the path of the bound gp130 peptide in the SOCS3 SH2 structure (Babon et al., 2006; Bergamin et al., 2006) but is oriented in an antiparallel fashion. Nonetheless, expected substrate interactions are formed by a phenylalanine that mimics the phosphotyrosine and a leucine residue that fills the hydrophobic +3 site.

SOCS SH2 domains show the greatest diversity in the conformations of the EF and BG loops that frame the hydrophobic +3 pocket, a region that shares little sequence identity between SOCS family members. The SOCS4 EF loop is three residues shorter than SOCS2/SOCS3 and arches away from the substrate pocket. In contrast, the longer EF loop in SOCS3 folds above the substrate pocket and makes significant contact with the bound gp130 peptide to contribute to the unusually high affinity of this interaction (Babon et al., 2006; Bergamin et al., 2006). The SOCS4 BG loop is well defined in contrast to the unstructured insertions that follow the αB helix in SOCS2 and SOCS3. In addition, the SOCS4 BG loop folds markedly inwards compressing the +3 site (Figures 2A and 2B). Overall, the binding surface of SOCS4 is distinguished by a strongly negative electrostatic surface potential in comparison to the mainly basic SOCS2 and SOCS3 (Figure 2C).

### Domain Organization Defines a Second Structural Subclass of SOCS Family Members

**Figure 3:**
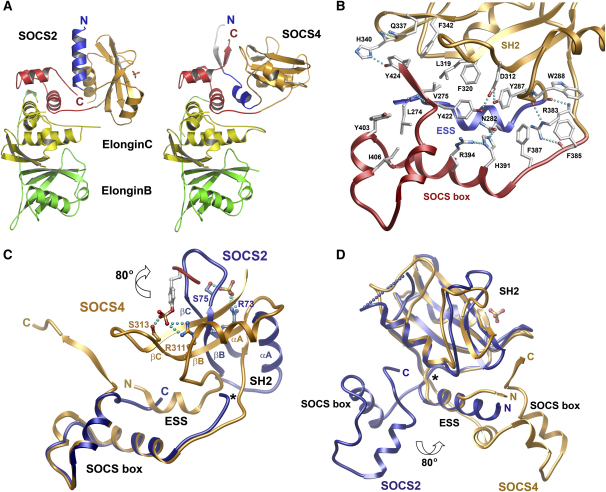
Alternative Domain Organization in the SOCS2 and SOCS4 Ternary Complexes (A) Comparison of the SOCS2-ElonginB/ElonginC and SOCS4-ElonginB/ElonginC structures highlighting the switch in packing between the SOCS2/SOCS4 C terminus and the N-terminal ESS helix (colored as in Figure 1). (B) Molecular interactions stabilizing the domain organization in SOCS4. (C) Structural overlay of the SOCS2 and SOCS4 SOCS box showing an 80° rotation of the SOCS4 SH2 domain relative to the SOCS2 SH2. The positions of the SOCS2 and SOCS4 phosphotyrosine pockets are indicated by a SOCS2-bound sulfate ion and a SOCS4-bound phosphotyrosine (modeled as in Figure 2). For clarity, the SOCS2 ESS is omitted and only the N-terminal half of each SH2 domain is shown. An asterisk denotes the position of the hinge point for rotation which occurs at R383/T384 in SOCS4. (D) Structural overlay of the SOCS2 and SOCS4 SH2 domains showing the alternative packing sites for the respective SOCS box domains on opposite faces of the ESS and SH2. The SOCS2-bound sulfate ion indicates the position of the SH2 phosphotyrosine pocket.

In SOCS2, the C terminus is buried in the core of the structure where it stabilizes the interface between the SH2 domain and the SOCS box (Bullock et al., 2006). A 14 residue C-terminal extension in SOCS4 prevents these interactions resulting in an alternative domain organization. Interestingly, the SOCS4 ESS helix replaces the SOCS2 C terminus in the domain interface (Figures 3A and 3B). This is accomplished by rotation of 80° of the SH2 domain with respect to SOCS2 (calculated by using the DynDom server [Hayward and Berendsen, 1998]).

An overlay of the two SOCS box-Elongin structures shows excellent superimposition up to a conserved threonine at the N-terminus of the SOCS box (T158 and T384 in SOCS2 and SOCS4, respectively) (Figures 3C and 3D). In both structures, this residue forms a hinge point where the backbone kinks to meet the SH2 domain. Significantly, the SOCS4 hinge has an insertion of R383, which redirects the path of the main chain. The alternative conformation is stabilized by the R383 side chain, which is buried in a hydrophobic pocket between the N-termini of the SH2 domain (βA residues Y287 and W288) and SOCS box (F385 and F387). The guanidinium group binds these structures together with hydrogen bonds to the main chain oxygens of Y287 and F385 (Figure 3B). The two phenylalanine side chains appear too large to be accommodated in a SOCS2-like conformation.

The main function of the C-terminal SOCS box extension seems to be the interaction with the SOCS4 N-terminus. These sequences are stabilized in an antiparallel β sheet that packs behind the SH2 DE loop (Figure 3A). The C-terminal residues of SOCS5–SOCS7 are more similar to SOCS4 than CIS1/SOCS1–SOCS3 and are also expected to pack on the surface of the SH2 domain. In particular Y424, which occupies the hydrophobic core of the C-terminal packing and hydrogen bonds to the DE loop, is strictly conserved in SOCS4–SOCS7, but this residue is not present in CIS1/SOCS1–3, giving additional support for two distinct domain packing interactions in SOCS family members.

### SOCS4 Is a High Affinity Binding Partner for EGFR pY^1092^

**Figure 4:**
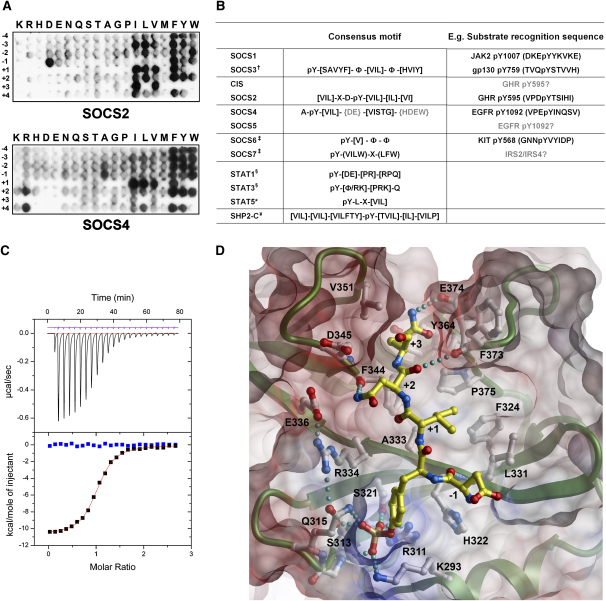
SOCS4 Is a High Affinity Binding Partner for EGFR pY^1092^ (A) OPAL membranes showing the substrate binding specificity of SOCS2 (top) and SOCS4 (below). Bound GST-SOCS-ElonginB/C complexes were detected by using anti-GST-HRP antibody. The large surface area of the SOCS complexes may contribute to the strong background binding to R/F/Y/W. Consequently, the preference for these residues is not considered here. (B) Comparison of the determined SOCS consensus recognition sequences and their suggested physiological targets. By sequence and functional similarity, the SOCS family members can be grouped into pairs as shown. Brackets ([ ]) indicate preferred amino acids, whereas gray braces ({ }) indicate nonpreferred amino acids. X denotes any amino acid, and Φ denotes any hydrophobic residue. †, De Souza et al. (2002); ‡, Krebs et al. (2002); ¥, Rodriguez et al. (2004); §, Wiederkehr-Adam et al. (2003); ^∗^, Barber et al. (2001). (C) The SOCS4 binding affinity for EGFR pY^1092^ was determined by ITC (K_D_ = 0.5 μM). Data for peptide titrations into SOCS4 are colored black, while data from a control experiment are colored blue and offset for clarity in the top panel. (D) Structural model of the SOCS4-EGFR complex. The complex was modeled by using the homologous SOCS3-gp130 crystal structure (PDB code: 2HMH) as a template peptide for EGFR, and side chains were optimized by using ICM-PRO (Abagyan et al., 1994). The SOCS4 F373 side chain (BG loop) is solvent exposed and was relaxed to open the +4 position. A complete model for the SOCS2-GHR complex could not be built because in the apo SOCS2 structure, the flexible EF loop is folded into the +2 site to block the path of the peptide. The preferred −1 aspartate can pack between SOCS2 K59 (αA6) and T93 (βD3) to maximize its hydrogen bonding potential and similar residues are present in CIS (R93 and T127). The SOCS2 hydrophobic selection at positions C-terminal to the phosphotyrosine can be understood from the presence of leucine at βD5, βE4, and BG3 (Bullock et al., 2006).

SOCS4 has been implicated in the regulation of EGFR degradation (Kario et al., 2005; Nicholson et al., 2005), but a direct interaction has not been demonstrated, and to date the substrate binding sites for SOCS4 have not been defined. To further delineate the activity of different SOCS family members, we used an oriented peptide array library (OPAL) (Rodriguez et al., 2004) to determine the sequence preferences of SOCS2 and SOCS4 and compared these data to previous analyses of SOCS3 (De Souza et al., 2002), SOCS6 (Krebs et al., 2002), and SOCS7 (Krebs et al., 2002) (Figure 4). The arrays presented represent, to our knowledge, the first study that used functionally expressed SOCS protein that has not required refolding. In common with most SH2 domains, the SOCS family exhibit sequence preferences at positions C-terminal to the phosphotyrosine and belong to a SH2 class selective for hydrophobic residues at the +1 and +3 positions. SOCS2 and SOCS4 show strong preference for isoleucine, leucine, and valine at both positions with the exclusion of leucine at +3 (Figure 4).

However, there are notable differences in substrate recognition between SOCS2/SOCS4 and classic SH2 domains like Src. In addition to residues C-terminal to the phosphotyrosine, SOCS2 and SOCS4 have preferred amino acids at positions N-terminal to the phosphotyrosine. For SOCS2, isoleucine, leucine and valine at the −3 and aspartate at the −1 position are selected, respectively. The SOCS2 SH2 consensus motif (Figure 4) is consistent with its binding to the pY^595^ site in growth hormone receptor (GHR), which is the suggested physiological target site for SOCS2 (Greenhalgh et al., 2005). Namely, the GHR pY^595^ peptide contains the preferred valine, aspartate, and isoleucine respectively at the −3, −1, and +3 positions, while the +1 threonine is a secondary selection site. In contrast, SOCS4 shows a strong preference for β-branched amino acids isoleucine, leucine, and valine at the +1 position and slight preference for isoleucine, leucine, and valine at the −3 and alanine at the −1 position. The selection against aspartate and glutamate at the +1 to +4 positions is consistent with the negative electrostatic surface potential of the SOCS4 substrate pocket and is a further determinant of SOCS4 specificity.

To date, only a limited number of SOCS target sites have been mapped, and these are largely restricted to substrates of CIS/SOCS1–SOCS3. We determined binding affinities for these previously identified sites in solution by using isothermal titration calorimetry (ITC). In addition, we analyzed the known target sites associated with EGFR degradation. Consistent with the OPAL data, SOCS2 was highly selective for the preferred growth hormone receptor site pY^595^ and no other targets were identified of similar affinity (Table 2). SOCS4 was found to bind to EGFR pY^1092^ (K_D_ = 0.5 μM) with an affinity comparable to the known physiological ligand Grb2 (K_D_ = 0.4–0.7 μM) (Chook et al., 1996; Lemmon et al., 1994). EGFR pY^1092^ contains a favorable valine at the −3 position and the preferred isoleucine at the +1 position, consistent with the determined SOCS4 SH2 consensus. In contrast, SOCS4 showed little affinity for the Cbl target site on EGFR pY^1069^ (K_D_ > 10 μM). Interestingly, SOCS4 also bound JAK2 pY^1007^ and KIT pY^568^ with an affinity below 3 μM, suggesting functional similarity with *Drosophila* SOCS36E (Callus and Mathey-Prevot, 2002; Rawlings et al., 2004) and human SOCS6 (Bayle et al., 2004), respectively. This affinity is similar to the binding of Grb7 to ErbB2 (K_D_ = 2.3 μM) (Ivancic et al., 2005). Other target sites bound with weaker affinity, particularly those with the loss of either the +1 or +3 hydrophobic position (Table 2). Overall, SOCS4-peptide interactions were characterized by a considerably larger enthalpic contribution to binding than SOCS2 suggesting the formation of a larger number of favorable polar contacts.

A structural model for the SOCS4-EGFR interaction was derived to understand the observed substrate specificity. The SOCS4 crystal packing (Figure 2B) supports a classic extended binding mode similar to the SOCS3-gp130 complex (Babon et al., 2006; Bergamin et al., 2006). SOCS4 has a highly unusual βD5 alanine (a position that frequently correlates with specificity (Songyang and Cantley, 1995) and consequently contains a substrate pocket lined with large hydrophobic side chains that fill the vacant packing. Here, the EGFR +1 isoleucine occupies a hydrophobic pocket formed between L331 (βD3) and F324, consistent with its primary selection in OPAL (Figure 4D). The BG loop packing compresses the substrate pocket so that EGFR makes close contact with the EF loop where the +2 asparagine can hydrogen bond to the backbone oxygen of F344. The +3 glutamine can extend to the back of the +3 pocket to interact with Y364 and E374. The OPAL selectivity for [IVSTG] at this position reflects the relatively small pocket volume and the larger glutamine residue may induce limited conformational strain. Overall, the SOCS4 specificity and binding mode is similar to the SHP2-IRS1 complex structure (PDB code: 1AYB) in which the extended IRS1 peptide contains a pYVNI sequence (Lee et al., 1994). The insulin receptor substrate protein family is a recognized target for SOCS1/SOCS3 (Rui et al., 2002) and SOCS6/SOCS7 (Krebs et al., 2002), and the close match with the SOCS4 consensus suggests that SOCS4 may also target members of this important protein family.

## Discussion

The SOCS family members SOCS4–SOCS7 were originally identified by their conserved SOCS box, an adaptor motif comprising three helices that associates substrate binding domains, such as the SOCS SH2 domain, ankryin, and WD40 repeats, with the ubiquitin ligase components ElonginC and ElonginB (Hilton et al., 1998). The crystal structures of the SOCS2 and SOCS4-ElonginB/ElonginC complexes highlight an important evolutionary divergence between the SOCS box of SOCS4–SOCS7 and that of CIS and SOCS1–SOCS3 (Bullock et al., 2006). The two families make alternative use of N- and C-terminal sequences to provide the SH2-SOCS box interdomain interface. By burying the C terminus in this interface the SOCS2-type domain organization partially exposes the ESS providing greater accessibility for the SOCS1/SOCS3 KIR domain. In contrast, SOCS4 buries the ESS between the SOCS box and SH2 domain to fulfill an equivalent packing role to the SOCS2 C terminus. The function of the SOCS4 C terminus is then redefined to stabilizing a new interface with the N terminus, which packs as an antiparallel β sheet behind the SH2 DE loop. The new domain organization correlates with the presence of a greatly expanded N-terminal domain, consisting of 300–400 residues, which is found in SOCS4–SOCS7, but not in other SOCS family members. This region remains to be structurally and functionally characterized. It is highly variable between the SOCS members and shows no similarity to domains of known three-dimensional structure.

**Figure 5:**
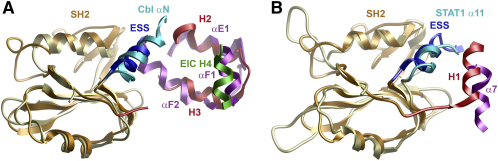
SOCS4 Interdomain Packing Is Similar to Cbl and STAT Family Proteins (A) The Cbl αN helix (cyan) is structurally equivalent to the SOCS4 ESS (blue), having similar length and position. This structure connects the Cbl SH2 (pale yellow) and EF-hand (purple) domains and adopts a similar position to the bound helical peptides in calmodulin structures. Further, the Cbl helices αE1, αF2, and αF1 are placed similarly to SOCS box H2, H3 (red), and ElonginC H4 (green), respectively, although they show alternative topology. The SOCS4 SH2 domain is colored orange. (B) The packing in Cbl has been likened to the STAT family in which the linker domain fulfills an equivalent packing role to the Cbl EF hand (Meng et al., 1999). Overlay of the STAT1 (pale yellow) and SOCS4 (orange) SH2 domains reveals structural similarity between the STAT1 α11 (cyan) and α7 helices (purple) and the SOCS4 ESS (blue) and H1 (red), respectively.

The SOCS box mediates further assembly with Cul5 and Rbx2 to form a RING-type E3 ubiquitin-ligase (Kamura et al., 1998, 2004). These complexes are proposed to function as stable scaffolds that present bound substrate to E2 enzymes with the correct distance and orientation for efficient ubiquitin transfer (Petroski and Deshaies, 2005; Zheng et al., 2002). An unexpected feature of the SOCS4 rearrangement is an 80° rotation of the ESS/SH2 domain with respect to SOCS2. This rotation does not affect the overall placement of the SH2 domain within the ternary complex, which is conserved with the substrate-binding domains of other cullin-dependent ubiquitin ligases. In particular, structural models for F box (Hao et al., 2007) and SOCS box (Figure S2, see the Supplemental Data available with this article online) complexes show a similar range of substrate peptide orientations suggesting that SOCS4 retains a viable scaffold to support E3 ligase activity. The new domain arrangement in SOCS4 also provides an interesting parallel with the packing found in the structures of Cbl (Meng et al., 1999) and STAT (Chen et al., 1998) family members revealing a common mechanism to stabilize SH2 structure. The SOCS4 packing is most closely related to the c-Cbl SH2 and EF hand domains (Figure 5). This similarity is intriguing given the interaction of both proteins with EGFR. This common arrangement appears to provide a more stable and rigid packing solution than the alternative SOCS2 structure, potentially reflecting the greater selection for rapid induction and degradation responses in the CIS/SOCS1–SOCS3 subfamily (for example, phosphorylation of the SOCS3 interface Y221 induces its degradation).

Cellular studies have shown induction of SOCS4 and SOCS5 upon EGF stimulation and subsequent SOCS box-dependent degradation of EGFR and inhibition of the mitogenic signal that is independent of Cbl (Kario et al., 2005; Nicholson et al., 2005). SOCS5 shows 84% sequence identity with SOCS4 within the region covered by this structure and has conserved residues at key sites determining substrate binding and domain orientation. The *Drosophila* ortholog SOCS36E shows similar conservation, and together these SOCS proteins form a tight subgroup within the SOCS family. SOCS5 immunoprecipitation of EGFR was inhibited by SH2 mutation, but the molecular basis for this interaction was not determined. We identified a high affinity binding site for SOCS4 at tyrosine 1092 in EGFR (K_D_ = 0.5 μM). This position is well characterized as an EGFR autophosphorylation site and is targeted with similar affinity by the SH2 domain of Grb2, which transduces EGF signaling. Immunoprecipitation studies supported a second constitutive EGFR interaction site within the SOCS5 N-terminal domain (Nicholson et al., 2005), and it is interesting to note that the N-terminus packs alongside the DE loop by the +2 pocket in the SOCS4 structure. This bidentate recognition would be reminiscent of the interaction of SOCS1 with JAK kinases, which also involves the KIR domain in addition to the SH2 domain. The Cbl and STAT SH2-substrate interactions also involve an extended binding interface with a second interaction domain (Mao et al., 2005; Meng et al., 1999). Such interaction is likely to further increase the SOCS-EGFR affinity as observed for the physiological Grb2/mSos1-EGFR complex (K_D_ = 0.3 μM) (Chook et al., 1996). SOCS5 function may differ from SOCS4 primarily by a further N-terminal extension that binds the box1 domain of the IL-4 receptor (IL-4Rα) to regulate STAT6 signaling in Th2 cell differentiation (Seki et al., 2002).

Multiple ubiquitin ligases have been identified for the ErbB family in addition to Cbl, which targets EGFR directly at pY^1069^ (K_D_ = 0.4 μM) or through Grb2 at pY^1092^, for example CHIP (ErbB2) (Xu et al., 2002), LNX1 (ErbB2) (Young et al., 2005), Nrdp1 (ErbB3) (Qiu and Goldberg, 2002), and AIP4/Itch (ErbB4) (Omerovic et al., 2007). Potentially, as inducible negative feedback inhibitors, the SOCS family may provide further regulation with alternative spatial and temporal control. Direct demonstration of SOCS4/SOCS5 ubiquitin ligase activity remains to be proven, but SOCS4/SOCS5 binding at EGFR pY^1092^ would nevertheless also compete directly with STAT3 consistent with reduced STAT3 signaling in cellular transfection studies (Kario et al., 2005). SOCS5 also reduced the levels of ErbB2 and ErbB4 (Kario et al., 2005), which have homologous sites to EGFR 1092 (pYINQ), for example pYVNQ (ErbB2) and pYLNP (ErbB4), while this motif is absent in ErbB3. From our existing peptide panel, we indeed detected tight SOCS4 binding to a similar pYINP site (K_D_ = 1.1 μM) (Table 2). In total there are 89 cytosolic tyrosines in the ErbB family, of which approximately half have been linked to signaling, with six Grb2 binding sites identified in EGFR alone (Schulze et al., 2005). Given its apparent relaxed specificity, SOCS4 appears similarly compatible with additional interaction sites, for example binding to EGFR pY^1138^ (pYLNT) would also be predicted. Further studies are required to delineate these activities and the physiological SOCS4 response.

Interestingly, SOCS2 is also suggested to downregulate STAT5b signaling from EGFR without degradation (Goldshmit et al., 2004), while SOCS1 and SOCS3 are reported to downregulate STAT1-EGFR signaling (Xia et al., 2002). Our OPAL specificity data support the strong association between SOCS2 and STAT5 target recognition sites, but do not suggest a simple correlation of one SOCS inhibitor regulating one STAT family member. Instead, we observe overlapping SOCS/STAT (and SHP2) sequence preferences rather than direct similarity. For example, in the EGFR Y^1092^ recognition site SOCS4 is selective for +1 isoleucine, whereas STAT3 is selective for +3 glutamine (Wiederkehr-Adam et al., 2003). Furthermore, Grb2 targets the same site with a β-hairpin binding mode that confers specificity for +2 asparagine (Songyang et al., 1993). These preferences highlight an important signaling control mechanism. Since the interactome of all three proteins is distinct, SOCS4 may inhibit a specific subset of STAT3 sites without perturbing other STAT3 targets that should remain active.

We also detected low micromolar binding of SOCS4 to JAK2 pY^1007^ consistent with studies on SOCS36E in *Drosophila* (Callus and Mathey-Prevot, 2002; Rawlings et al., 2004). Previous work has noted a degree of sequence conservation between the KIR domain of SOCS1/SOCS3 and the equivalent region of SOCS4/SOCS5 (and SOCS36E) (Narazaki et al., 1998; Nicholson et al., 1999). The SOCS1 KIR domain has activity against both the JAK2 and EGFR kinase domains (Waiboci et al., 2007), but similar SOCS4/SOCS5 activity has not been demonstrated due to their low expression levels in these studies (Nicholson et al., 1999). While these experiments merit further investigation, the data presented here reveal a different KIR structural environment in SOCS4/SOCS5 and provide an alternative mechanism for the observed SOCS4/SOCS5 inhibition of EGFR through binding at pY^1092^. These structural and specificity data open new opportunities to rapidly characterize the extended SOCS4–SOCS7 subfamily further.

## Experimental Procedures

### Protein Expression and Crystallization

Human SOCS4 (amino acids 274–437), ElonginC (amino acids 17–112), and ElonginB were coexpressed in BL21(DE3) from the plasmids pNIC-SOCS4 and pACYCDUET-ElCB. Ternary complex was purified by nickel-affinity, size-exclusion, and anion-exchange chromatography and concentrated to 11 mg/ml in 50 mM HEPES (pH 7.5), 250 mM NaCl, 10 mM DTT. The N-terminal hexahistidine tag was buried in the structure and therefore protected from cleavage. The protein complex was judged to be at least 95% pure by SDS PAGE, and the correct molecular weight of all three proteins was confirmed by using liquid chromatography electrospray ionization mass spectrometry. Crystals were grown at 4°C in 150 nl sitting drops by using a mother liquor of 10% PEG6000 and 2M NaCl.

### Structure Determination

SOCS4 diffraction data were collected on a frozen crystal (100 K) at the Swiss Light Source Beamline 10 (Villigen, Switzerland). Images were indexed and integrated with MOSFLM and scaled with SCALA within the CCP4 program suite (CCP4, 1994). The structure was solved by molecular replacement with the SOCS2-ElonginB/ElonginC complex (PDB code: 2C9W) as a search model with the program PHASER (Storoni et al., 2004). Iterative rounds of rigid-body refinement and restrained refinement with TLS (translation-libration-screw) against maximum likelihood targets were interspersed by manual rebuilding of the model with COOT (Emsley and Cowtan, 2004) and XFIT/XTALVIEW (McRee, 1999; Murshudov et al., 1997). Figures were prepared with PYMOL (DeLano, 2002) and ICM-PRO (Abagyan et al., 1994).

### Isothermal Titration Calorimetry

Experiments were carried out in 50 mM HEPES (pH 7.5), 150 mM NaCl, 1 mM DTT at 20°C, injecting 0.3–0.4 mM peptide solution into 15 μM protein solution. Blank titrations were subtracted from binding data, and data were processed by using ORIGIN software provided with the instrument. Peptides: EGFR pY1069 LQR(pY)SSDPTGA; EGFR pY1092 PVPE(pY)INQSVP; EpoR pY402 ASFE(pY)TILDPS; GHR pY487 NIDF(pY)AQVSDIT; GHR pY595 PVPD(pY)TSIHIV; gp130 pY759 STVQ(p)YSTVVHS; JAK2 pY1007 QDKE(pY)YKVKEPG; KIT pY568 NGNN(pY)VYIDPT; LeptinR pY1077 KSVC(pY)LGVTSVN; Peptide pYINP TLDN(pY)INPDAA.

### Determination of SOCS Substrate Binding Specificity Using OPAL

Oriented peptide array libraries (OPAL) were synthesized and screened as previously described (Rodriguez et al., 2004). Briefly, the synthesized OPAL has the sequence AXXXX[pY]XXXXA, where X is a mixture of 19 amino acids (except Cys). The OPAL membrane was first blocked with 5% milk in TBST (0.1M Tris-HCl [pH 7.4], 150 mM NaCl, 0.1% Tween20) for one and half hours. GST-SOCS-ElonginB/ElonginC complex fusion proteins (1 μg) were incubated with anti-GST-HRP (0.2 μg, Amersham) for 1 hr and then added to the array membrane at a final concentration of 0.5 μg/ml for 1 hr. The array membrane was subsequently washed three times with TBST for 10 min, and positive peptide spots were visualized by ECL.

## Figures and Tables

**Table 1:** Crystallographic Data and Refinement Statistics

Data Collection	SOCS4-ElonginC-ElonginB		
Space group	H3		
Cell dimensions (Å)	a = 154.770, b = 154.770, c = 67.909		
Resolution (Å)	2.55		
Total obs. (unique, red.)	56643 (19724, 2.71)		
Completeness (outer shell^a^)	99.7% (97.9%)		
R_merge_ (outer shell^a^)	0.13 (0.48)		
I/σ (outer shell^a^)	9.14 (2.0)		

Refinement			

R_work_ (R_free_^b^) (%)	17.4 (22.3)		
Protein atoms (water)	2806 (113)		
Hetero groups:	ethylene glycol, Na^+^, Cl^−^		
Rmsd bond length (Å)	0.013		
Rmsd bond angle (^o^)	1.438		

Average B Factor (Å^2^)			

Protein atoms	29.8 (SOCS4) 34.2 (El.B) 32.1 (El.C)		
Solvent atoms	26.1		
Other	53.7		

Ramachandran	SOCS4	ElonginB	ElonginC

Allowed (%)	100	96.7	100
Generously allowed (%)	0	2.2	0
Dissallowed (%)	0	1.1	0

a Outer shell 2.65–2.55 Å.

b Using randomly selected 5% of data.

**Table 2:** ITC Binding Data for Phosphotyrosine Peptides

Peptide	K_D_ (μM)	K_B_ × 10^5^ (M^−1^)	Δ*H*^obs^ (kcal/mol)	*T*Δ*S* (kcal/mol)	Δ*G* (kcal/mol)	N^a^
SOCS2/ElonginBC						

EGFR pY1069	>15					
EGFR pY1092	>15					
EpoR pY402^b^	7.1	1.42 ± 0.27	−3.69 ± 0.46	3.34	−7.03	0.73
GHR pY487	11.3	0.89 ± 0.64	−1.49 ± 0.76	5.16	−6.65	1.05
GHR pY595^b^	1.6	6.08 ± 1.03	−3.35 ± 0.11	4.54	−7.89	1.22
gp130 pY759^c^	>15					
JAK2 pY1007	8.1	1.23 ± 0.34	−3.62 ± 0.63	3.22	−6.84	0.89
KIT pY568	8.3	1.20 ± 0.23	−3.62 ± 0.38	3.19	−6.81	1.12
LeptinR pY1077	>15					
Peptide pYINP	>15					

SOCS4/ElonginBC						

EGFR pY1069	11.6	0.86 ± 0.12	−13.0 ± 2.0	−6.35	−6.64	0.95
EGFR pY1092	0.5	19.0 ± 1.0	−10.8 ± 0.1	−2.36	−8.42	1.00
EpoR pY402	7.7	1.30 ± 0.16	−14.5 ± 1.3	−7.59	−6.86	0.71
GHR pY487	5.1	1.96 ± 0.11	−10.6 ± 0.2	−3.49	−7.10	1.18
GHR pY595	6.1	1.63 ± 0.18	−10.1 ± 0.5	−3.08	−6.99	0.96
gp130 pY759^c^	7.1	1.41 ± 0.17	−7.08 ± 0.40	−0.17	−6.91	1.12
JAK2 pY1007	2.9	3.43 ± 0.25	−11.3 ± 0.2	−3.87	−7.41	1.00
KIT pY568	2.9	3.43 ± 0.24	−7.03 ± 0.12	0.40	−7.43	1.29
LeptinR pY1077	5.5	1.82 ± 0.13	−7.69 ± 0.24	−0.63	−7.06	0.96
Peptide pYINP	1.1	9.40 ± 0.47	−12.2 ± 0.1	−4.16	−8.03	0.79

a Stoichiometry and curve fitting errors determined from a single binding site model with the Microcal Origin software.

b Bullock et al., 2006.

c Note the human numbering differs from murine gp130 pY757. The preferred substrate peptide is shown in bold. ITC binding curves for these data are shown in Figure S1.

## Accession Numbers

Atomic coordinates and structure factors have been deposited in the Protein Data Bank (PDB code: 2IZV).

## Supplemental Data

Document S1. Two Figures
