# Supplementary material for: Structure of the SOCS4-ElonginB/C Complex Reveals a Distinct SOCS Box Interface and the Molecular Basis for SOCS-Dependent EGFR Degradation
Source: Structure. 2007 Nov 13;15(11):1493–504. doi: 10.1016/j.str.2007.09.016 (PMC2225448; doi:10.1016/j.str.2007.09.016)
Supplement: Document S1. Two Figures [file mmc1.pdf]

Structure *15*

Supplemental Data

Structure of the SOCS4-ElonginB/C Complex Reveals a Distinct SOCS Box Interface and the Molecular Basis for SOCS-Dependent EGFR Degradation

Alex N. Bullock, Maria C. Rodriguez, Judit É. Debreczeni, Zhou Songyang, and Stefan Knapp

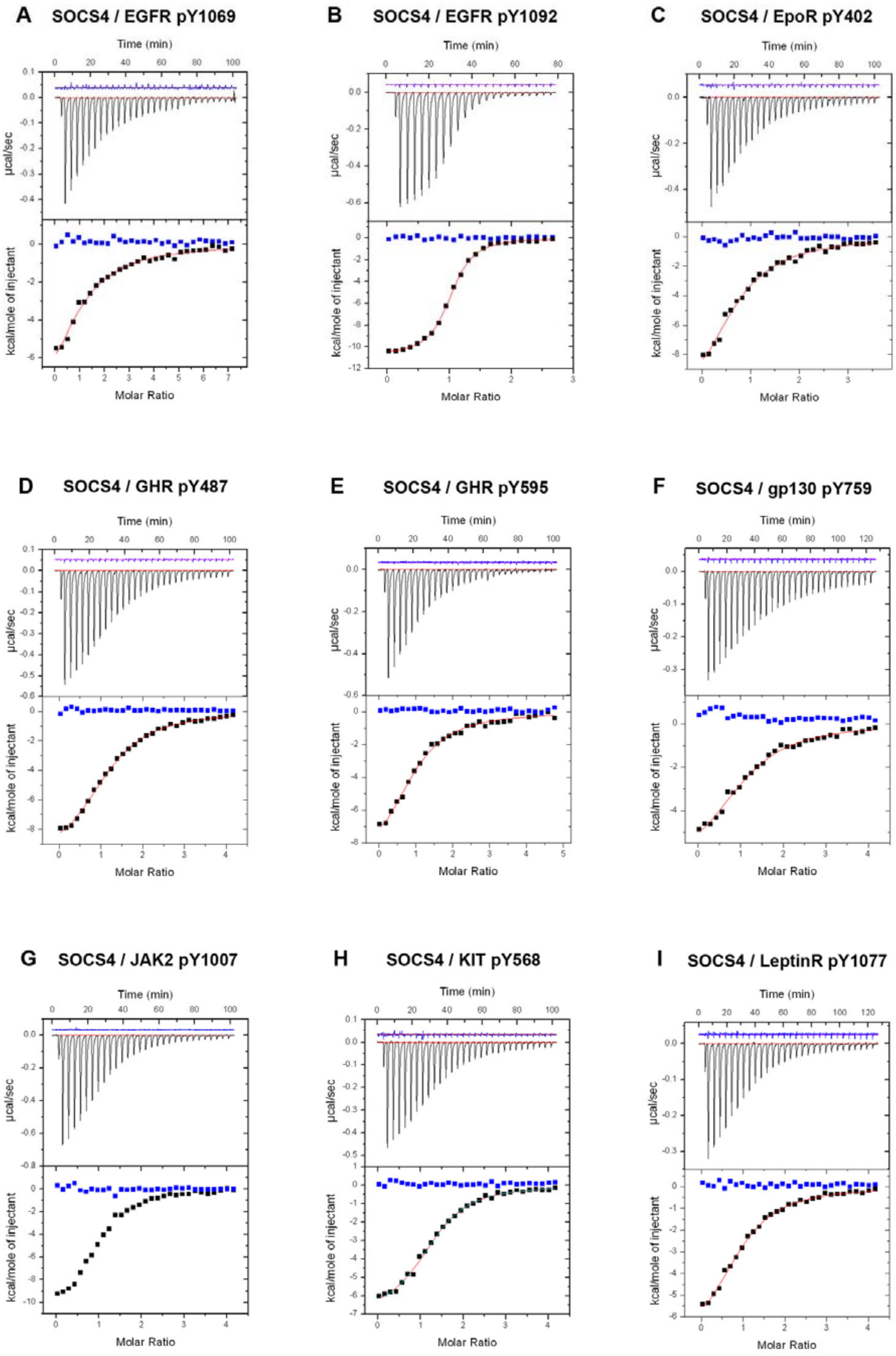
A SOCS4 / EGFR pY1069
B SOCS4 / EGFR pY1092
C SOCS4 / EpoR pY402
D SOCS4 / GHR pY487
E SOCS4 / GHR pY595
F SOCS4 / gp130 pY759
G SOCS4 / JAK2 pY1007
H SOCS4 / KIT pY568
I SOCS4 / LeptinR pY1077
Time (min)
µcal/sec
kcal/mole of injectant
Molar Ratio

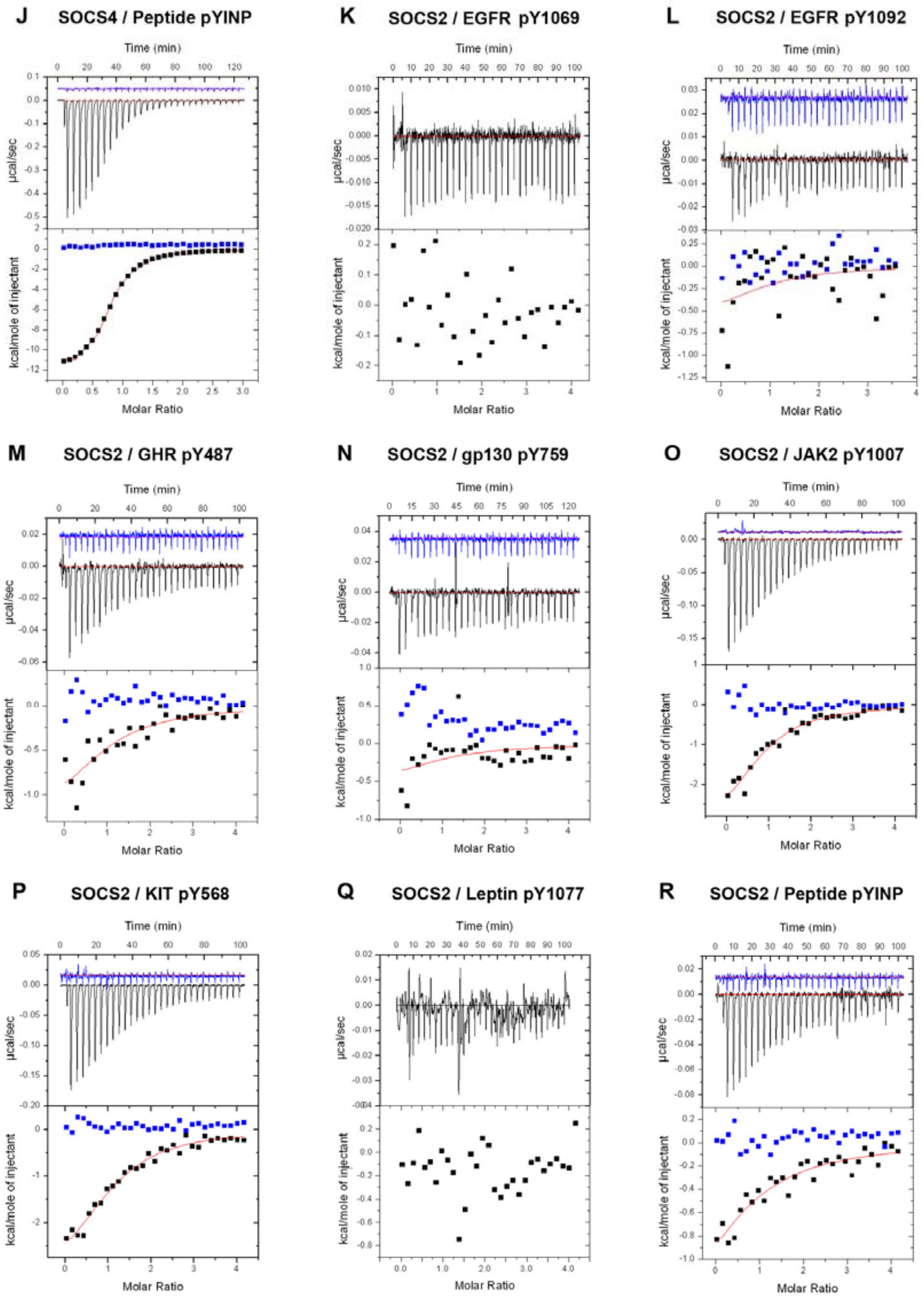

Figure S1. ITC binding curves for SOCS2 and SOCS4-ElonginBC complexes with the indicated peptide substrates.

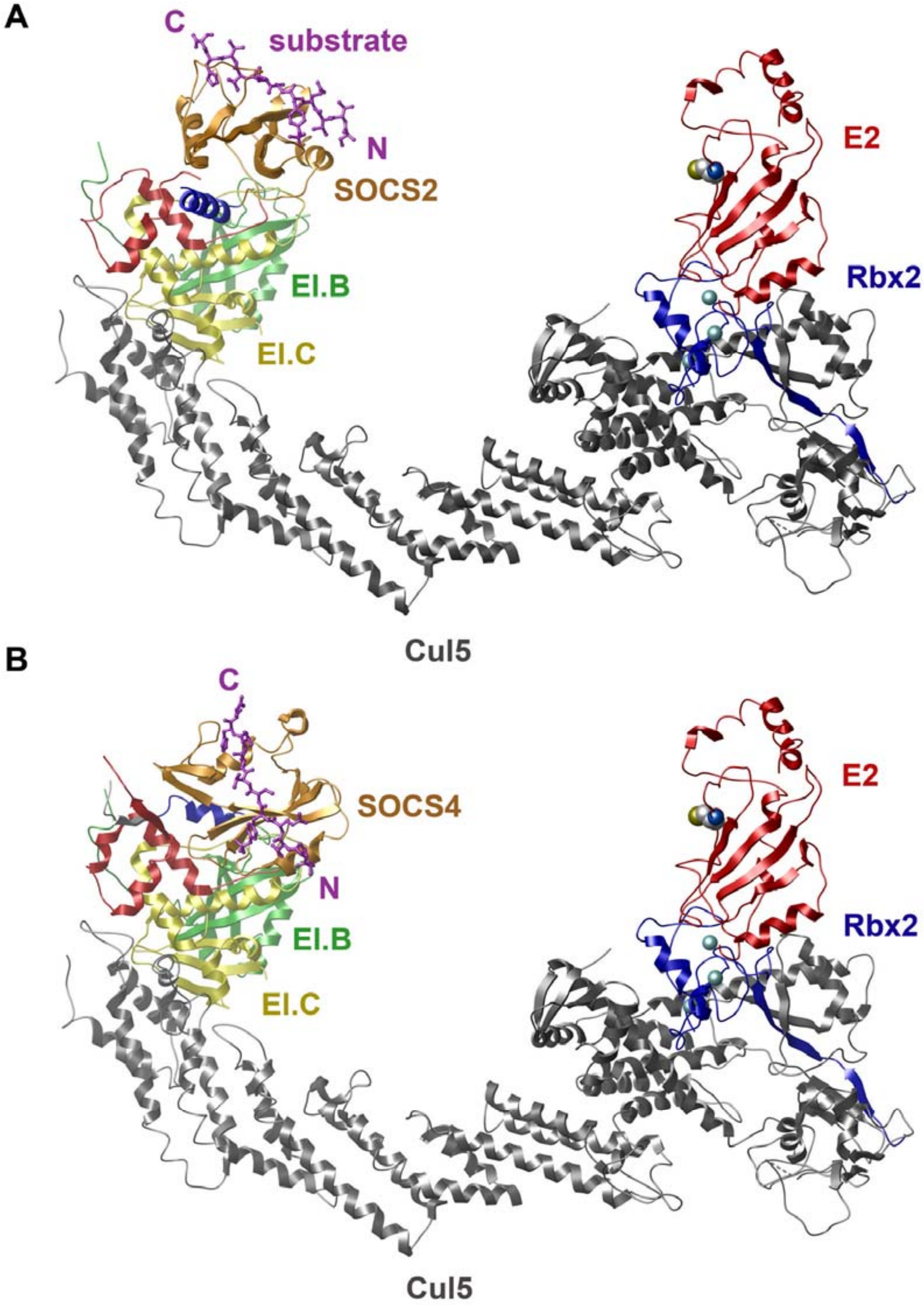

Figure S2. Models for the SOCS2 and SOCS4 ubiquitin ligase complexes. The Cul5 complexes were modelled as described previously (Bullock et al.2006) using a substrate peptide superimposed from the structure of the SOCS3-gp130 complex (Bergamin et al. 2006). The E2 active site cysteine which is conjugated to ubiquitin is shown in space fill.
